# Confronting the economic burden of diabetes: Can TCM DRG payment reform in China offer a viable solution? Insights from empirical research in Western China

**DOI:** 10.3389/fpubh.2026.1792742

**Published:** 2026-03-12

**Authors:** Meng-en Chen, Haojia Hou, Jingyu Yang, Fanxin Kong, Tianzhen Cong, Qian Zhang

**Affiliations:** 1 School of Traditional Chinese Medicine, Beijing University of Chinese Medicine, Beijing, China; 2 School of Management, Beijing University of Chinese Medicine, Beijing, China; 3 School of Health Management, Gansu University of Chinese Medicine, Lanzhou, China; 4 Gansu Health and Population Development Research Center, Health Commission of Gansu Province, Lanzhou, China; 5 Jining Medical University, Jining, China; 6 Department of General Affairs, Sichuan Clinical Research Center for Cancer, Sichuan Cancer Hospital & Institute, Sichuan Cancer Center, University of Electronic Science and Technology of China, Chengdu, China

**Keywords:** diabetes, DRG, hospitalization costs, length of stay, TCM

## Abstract

**Background:**

Diabetes poses a major global public health challenge, carrying significant economic implications worldwide. In China, the ongoing implementation of Diagnosis Related Groups (DRG) payment reforms, especially within Traditional Chinese Medicine (TCM) contexts, is critical in improving diabetes patient care and alleviating associated economic burdens.

**Methods:**

We examined 2,804 hospitalized diabetes patients at Qingyang City Hospital of Chinese Medicine in Gansu Province from 2017 to 2022. Using univariate and interrupted time-series (ITS) analyses, we compared patient visit data, healthcare-related costs, and length of stay pre- and post-DRG reform.

**Results:**

Following DRG reform at Qingyang City Hospital of Chinese Medicine, significant differences were noted in patients’ age, visit times, type of diabetes, complications and comorbidities, use of Chinese medicine diagnostic and therapeutic equipment, and surgeries and operations, compared with the pre-reform period (*p* < 0.05). Post-reform, there was a noteworthy decrease in hospitalization cost and Western medicine cost, and TCM treatment cost (*p* < 0.05), while Chinese medicine cost remained stable but the overall cost level increased (*p* > 0.05). Additionally, there was a slight reduction in length of stay after the reform, although this change did not reach statistical significance (*p* > 0.05).

**Conclusion:**

DRG reform significantly reduces hospitalization cost, TCM treatment cost, and Western medicine cost for diabetes patients in TCM hospitals. However, its impact on Chinese medicine cost and length of stay is limited. Future reforms should capitalize on the unique strengths of TCM treatment, enhance cost management strategies, and focus on minimizing length of stay and medical expenses while ensuring effective patient care.

## 1 Introduction

Diabetes has emerged as one of the most pressing public health challenges worldwide (1). According to the International Diabetes Federation (IDF), the number of diabetes patients globally exceeds 500 million and is projected to surpass 700 million by 2045 (2, 3). The high costs associated with diabetes treatment impose a significant economic burden on patients and their families, with global healthcare expenditures reaching nearly $760 billion in 2019 and anticipated to exceed $800 billion by 2045 (2, 4). Additionally, complications arising from diabetes further strain healthcare systems across countries (5–8).

China has the largest number of diabetes patients, with a prevalence rate of approximately 11%, accounting for nearly one-quarter of the global diabetes population (9, 10). By 2030, the number of diabetes patients in China is expected to exceed 140 million. The ongoing rise in diabetes cases, exacerbated by an aging population, poses substantial challenges to healthcare services and increases the economic burden of the disease in the country (11–13). In fact, the delivery of healthcare services for patients with chronic conditions, including diabetes, has become a prominent area of concern for the Chinese government.

TCM not only has a long history of inheritance, but also has unique treatment methods, with the characteristics of being “simple, convenient, experimental, and inexpensive”, providing distinct advantages in the prevention and treatment of various diseases (14–16). Recent studies have demonstrated that TCM offers significant advantages in blood glucose control and in alleviating complications, thereby enhancing patients’ quality of life (17–19). Consequently, optimizing the role of TCM hospitals in diabetes management is critical for improving treatment outcomes and reducing healthcare costs in China.

DRG classifies cases based on clinical symptoms and resource utilization (20), aiming to encourage healthcare institutions to enhance service efficiency and quality through refined management and rational payment methods, ultimately reducing healthcare costs (21–23). Of course, the DRG reform also has a significant impact on the clinical treatment outcomes for patients (24, 25). In response to escalating medical expenses, China is advancing comprehensive DRG-based health insurance payment reform, with plans for full implementation in hospitals by 2025 (26, 27). Given its integral role in the Chinese healthcare system, TCM is a key focus of the DRG reform.

The DRG payment reform in TCM hospitals aims not only to control hospitalization cost but also to leverage the distinctive advantages of TCM, providing more efficient and cost-effective treatment options for diabetes and other conditions where TCM excels. Against this backdrop, our study investigated the impact of DRG payment reform on healthcare-related costs and length of stay for diabetes patients at the TCM hospital in Qingyang City, Gansu Province. This study aims to offer insights and strategies for enhancing the DRG reform in China, emphasizing the distinctive contributions of TCM in alleviating the economic burden associated with chronic diseases such as diabetes.

## 2 Methods

### 2.1 Study design

The implementation of DRG-based payment reform at Qingyang Hospital of Traditional Chinese Medicine has introduced a case-mix bundled payment model for inpatient services across the entire hospital, replacing the previous fee-for-service and other charging methods. This transition has played a notable role in curbing the excessive growth of hospitalization costs and optimizing the utilization of medical resources. To effectively assess the impact of DRG reform on healthcare-related costs and length of stay for diabetes patients in TCM hospitals, this study utilized a quasi-experimental interrupted time-series (ITS) model to analyze data from the TCM hospital in Qingyang City, Gansu Province, China. As the first tertiary TCM hospital in Western China to implement DRG payment reform, this research is crucial for enhancing and refining DRG health insurance payment strategies within the TCM framework.

This single-center, uncontrolled ITS study utilized data obtained from the Health Commission of Gansu Province, encompassing hospital records from Qingyang City Hospital of Chinese Medicine spanning January 2017 to June 2022. Inclusion criteria were based on the International Classification of Diseases (ICD)-10 codes (E10-E14) and TCM codes BNV060 (1995 edition) or A06.09 (2021 edition). Exclusion criteria included the length of stay of less than 1 day or more than 90 days, hospitalization cost of zero, and any visit information that could not be verified.

Based on the specified inclusion and exclusion criteria, a total of 2,804 records were included in the analysis. The dataset comprised patient demographic information, visit details, and healthcare-related costs and length of stay. Demographic data included gender, age, and nationality, while medical information encompassed visit time, types of diabetes, complications and comorbidities, etc. Healthcare-related costs included hospitalization cost, TCM treatment cost, Chinese medicine cost, and Western medicine cost.

### 2.2 Statistical analysis

Starting in October 2019, Qingyang City in Gansu Province initiated a trial of the DRG payment system. For this study, the period from January 2017 to September 2019 was designated as the pre-reform stage, while the post-reform stage spanned from October 2019 to June 2022. To ensure a robust analysis of health economic costs, this study employed 2016 as the base year and adjusted the relevant costs using the Consumer Price Index (CPI) for healthcare costs in Gansu Province from 2017 to 2022, thereby minimizing potential research bias.

We conducted a comparative analysis of patient visit characteristics pre- and post-DRG reform. For normally distributed continuous variables, paired sample *t*-tests were utilized, with data presented as means and standard deviations. For non-normally distributed continuous variables, the Wilcoxon rank-sum tests were applied, and results were expressed as median and quartiles. Chi-square tests were employed for categorical variables to assess changes pre- and post-DRG reform, with data reported as frequencies and percentages. Subsequently, we utilized ITS analysis to examine core healthcare-related costs indicators (hospitalization cost, TCM treatment cost, Chinese medicine cost, and Western medicine cost) and length of stay among diabetes patients in TCM hospitals, aiming to investigate improvements in these indicators following the DRG reform. For the purpose of this study, the four cost categories were defined as follows: hospitalization cost refers to the total direct medical expenses incurred during a single inpatient episode, serving as a core indicator of disease economic burden and healthcare resource consumption. TCM treatment cost specifically denotes charges for non-pharmaceutical therapies delivered through appropriate TCM techniques, such as acupuncture, moxibustion, and tuina. Chinese medicine cost encompasses expenditures on medicinal substances, including Chinese herbal pieces, proprietary Chinese medicines, and TCM preparations. Western medicine Cost, meanwhile, comprises expenses related to chemical drugs and biological agents.

This study applied an ITS model, a quasi-experimental approach widely used in public health and healthcare policy research (28–30). The model equation is:


$$Y_{t}=\beta_{0}+\beta_{1}T_{t}+\beta_{2}X_{t}+\beta_{3}X_{t}T_{t}+\beta_{4}X_{\text{Covid}-19}+\varepsilon_{t}$$

**Figure 1:**
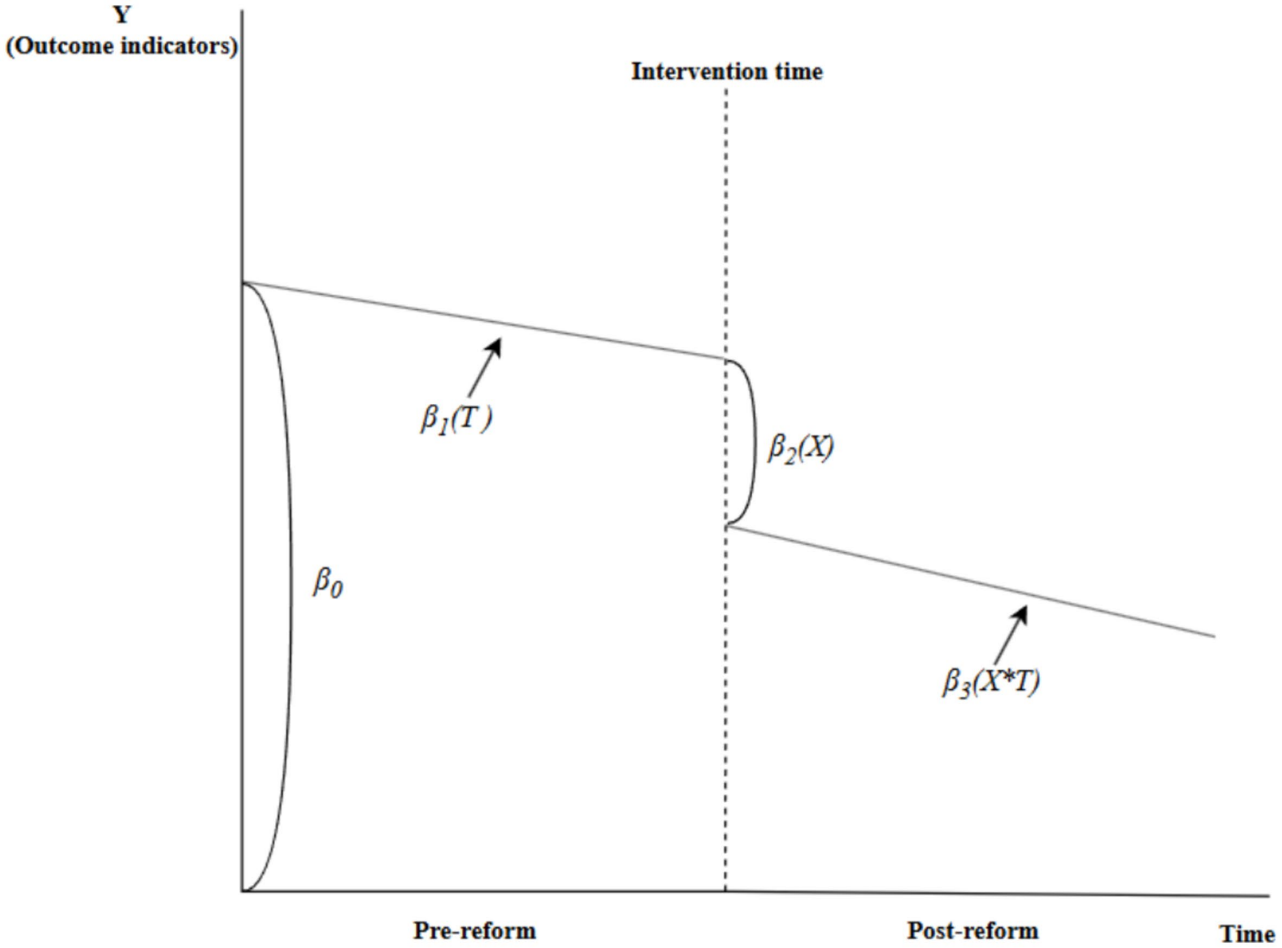
Schematic diagram of ITS model.

In this equation, *Y*_t_ is the dependent variable, representing the primary outcome. *β*_0_ is the intercept, *β*_1_ indicates the pre-reform trend (slope), *β*_2_ reflects the level change at the time of reform, and *β*_3_ represents the change in slope after the reform, with the post-reform slope being “*β*_1_ + *β*_3_”. *β*_4_ denotes the coefficient of the COVID-19 control variable. *X*_t_ and *X*_Covid-19_ are dummy variables representing the DRG reform and the COVID-19 pandemic, respectively. Specifically, *X*_t_ takes a value of “0” before the reform and “1” after, while *X*_Covid-19_ takes a value of “0” before the pandemic and “1” after. Besides, *T*_t_ represents the time variable spanning 66 consecutive months from January 2017 to June 2022. The interaction term is denoted as *X*_t_*T*_t_, with *ε*_t_ representing the random error. A schematic representation of the model is provided in Figure 1.

Due to the skewed nature of the data distribution for both healthcare-related expenditures and length of stay, the monthly median values were used as they provide a more representative measure, thereby ensuring analytical robustness and objectivity. The Cumby-Huizinga tests were used to detect autocorrelation in the dependent variable, with adjustments made using the “lag (#)” command and the Newey-West method (31–33). All statistical data organization and analysis were performed using EXCEL 2019, SPSS 26.0, and Stata 15.0, with the significance level set at *α* = 0.05.

## 3 Results

### 3.1 General information on hospitalized patients at Qingyang City Hospital of Chinese Medicine pre- and post-DRG reform

As shown in Table 1, there were no statistical differences in gender, nationality, use of Chinese medicine diagnostic and treatment techniques, and diagnosis and treatment based on Chinese medicine evidence among patients pre- and post-DRG reform (*p* > 0.05), but there were significant differences in age, visit times, type of diabetes, complications and comorbidities, use of Chinese medicine diagnostic and therapeutic equipment, surgeries and operations, hospitalization cost, TCM treatment cost, Chinese medicine cost, Western medicine cost and length of stay among patients pre- and post-DRG reform (*p* < 0.05).

**Table 1:** General information on diabetic patients pre- and post-DRG reform.

Items	TCM DRG reform		*Z*/*χ*^2^-value	*P*-value
	Pre-reform (*n* = 1,104)	Post-reform (*n* = 1700)		
Male, No. (%)^a^^,^^f^	740 (67.03)	1,118 (65.76)	0.479	0.489
Age, M (Q1, Q3), y^g^	56 (48,67)	58 (50,67)	−14.159	<0.001
Han, No. (%)^b^^,^^f^	1,102 (99.82)	1,699 (99.94)	0.142	0.706
One time, No. (%)^c^^,^^f^	1,068 (96.74)	1,343 (79.00)	174.772	<0.001
Type 2 diabetes mellitus (Yes), No. (%)^d^^,^^f^	737 (66.76)	1,217 (71.59)	7.395	<0.001
Complications and comorbidities (Yes), No. (%)^e^^,^^f^	1,102 (99.82)	877 (51.59)	747.473	<0.001
Use of Chinese medicine diagnostic and therapeutic equipment (Yes), No. (%)^e^^,^^f^	707 (64.04)	1,191 (70.06)	11.087	0.001
Use of Chinese medicine diagnostic and treatment techniques (Yes), No. (%)^e^^,^^f^	787 (71.29)	1,265 (74.41)	3.332	0.068
Diagnosis and treatment based on Chinese medicine evidence (Yes), No. (%)^e^^,^^f^	796 (72.10)	1,274 (74.94)	2.793	0.095
Surgeries and operations (Yes), No. (%)^e^^,^^f^	1,083 (98.10)	793 (46.65)	800.201	<0.001
Hospitalization cost, M (Q1, Q3), CNY^g^	5291.01 (4154.37, 6535.69)	5055.7 (3966.12, 6408.00)	−2.451	0.014
TCM treatment cost, M (Q1, Q3), CNY^g^	528.36 (337.58, 711.83)	132.88 (91.99, 205.76)	−24.053	<0.001
Chinese medicine cost, M (Q1, Q3), CNY^g^	522.59 (255.54, 813.61)	653.16 (351.9, 980.51)	−5.531	<0.001
Western medicine cost, M (Q1, Q3), CNY^g^	1619.43 (1086.67, 2307.46)	890.96 (522.81, 1442.71)	−15.159	<0.001
Length of stay, M (Q1, Q3), d^g^	12.00 (9.00, 14.00)	11.00 (8.00, 14.00)	−2.013	0.044

TCM, traditional Chinese medicine; DRG, diagnosis-related group; M (Q1, Q3), Median (First Quartile, Third Quartile); CNY, Chinese Yuan.

a Sex: male and female.

b Nationality: Han and other nationalities.

c Visit time: one time and two or more times.

d Type of diabetes: type 2 diabetes mellitus (the main types of diabetes in China) and other types of diabetes mellitus.

e Item categorization includes yes and no.

f The categorical data were presented as numbers (frequencies, %), Chi-square test was used for categorical data (χ^2^).

g The non-normal distribution continuous data were presented as “median (the first quartile, the third quartile)”, the data were compared using Wilcoxon rank sum test (Z).

### 3.2 Results of ITS analysis of DRG reform on healthcare-related costs at Qingyang City Hospital of Chinese Medicine

We performed Cumby-Huizinga autocorrelation tests on hospitalization cost, TCM treatment cost, Chinese medicine cost, and Western medicine costs at Qinyang Hospital. The results revealed that hospitalization cost may exhibit first-order autocorrelation, while TCM treatment cost, Chinese medicine cost, and Western medicine cost may show second-order autocorrelation, as detailed in Table 2. To ensure the validity of the ITS analysis, we adjusted for autocorrelation by employing the “lag (1)” and “lag (2)” commands in the analysis of healthcare-related costs.

**Table 2:** Autocorrelation tests results of healthcare-related costs for diabetic patients.

Items	*H*_0_: q = 0 (serially uncorrelated)				*H*_0_: q = lag-1				Items	*H*_0_: q = 0 (serially uncorrelated)				*H*_0_: q = lag-1			
	*H*_1_: s.c. present at range specified				*H*_1_: s.c. present at lag specified					*H*_1_: s.c. present at range specified				*H*_1_: s.c. present at lag specified			
	lags	chi2	df	*p*-value	lags	chi2	df	*p*-value		lags	chi2	df	*P*-value	lags	chi2	df	*P*-value
Hospitalization cost	1-1	14.87	1	<0.001	1	14.87	1	<0.001	TCM treatment cost	1-1	35.91	1	<0.001	1	35.91	1	<0.001
	1-2	14.89	2	0.001	2	2.02	1	0.155		1-2	36.45	2	<0.001	2	14.11	1	<0.001
	1-3	18.79	3	<0.001	3	0.60	1	0.440		1-3	36.63	3	<0.001	3	2.43	1	0.119
	1-4	20.69	4	<0.001	4	4.15	1	0.042		1-4	36.68	4	<0.001	4	0.38	1	0.539
	1-5	21.73	5	0.001	5	5.62	1	0.018		1-5	37.27	5	<0.001	5	0.06	1	0.815
	1-6	21.78	6	0.001	6	1.69	1	0.194		1-6	37.77	6	<0.001	6	0.07	1	0.796
Chinese medicine cost	1-1	6.71	1	0.010	1	6.71	1	0.010	Western medicine cost	1-1	34.29	1	<0.001	1	34.29	1	<0.001
	1-2	10.10	2	0.006	2	5.48	1	0.019		1-2	38.07	2	<0.001	2	4.15	1	0.042
	1-3	10.64	3	0.014	3	0.23	1	0.633		1-3	39.89	3	<0.001	3	0.01	1	0.922
	1-4	11.03	4	0.026	4	0.00	1	0.977		1-4	40.51	4	<0.001	4	2.62	1	0.106
	1-5	14.56	5	0.012	5	3.44	1	0.064		1-5	40.80	5	<0.001	5	5.27	1	0.022
	1-6	15.01	6	0.020	6	2.33	1	0.127		1-6	41.26	6	<0.001	6	5.04	1	0.025

**Figure 2:**
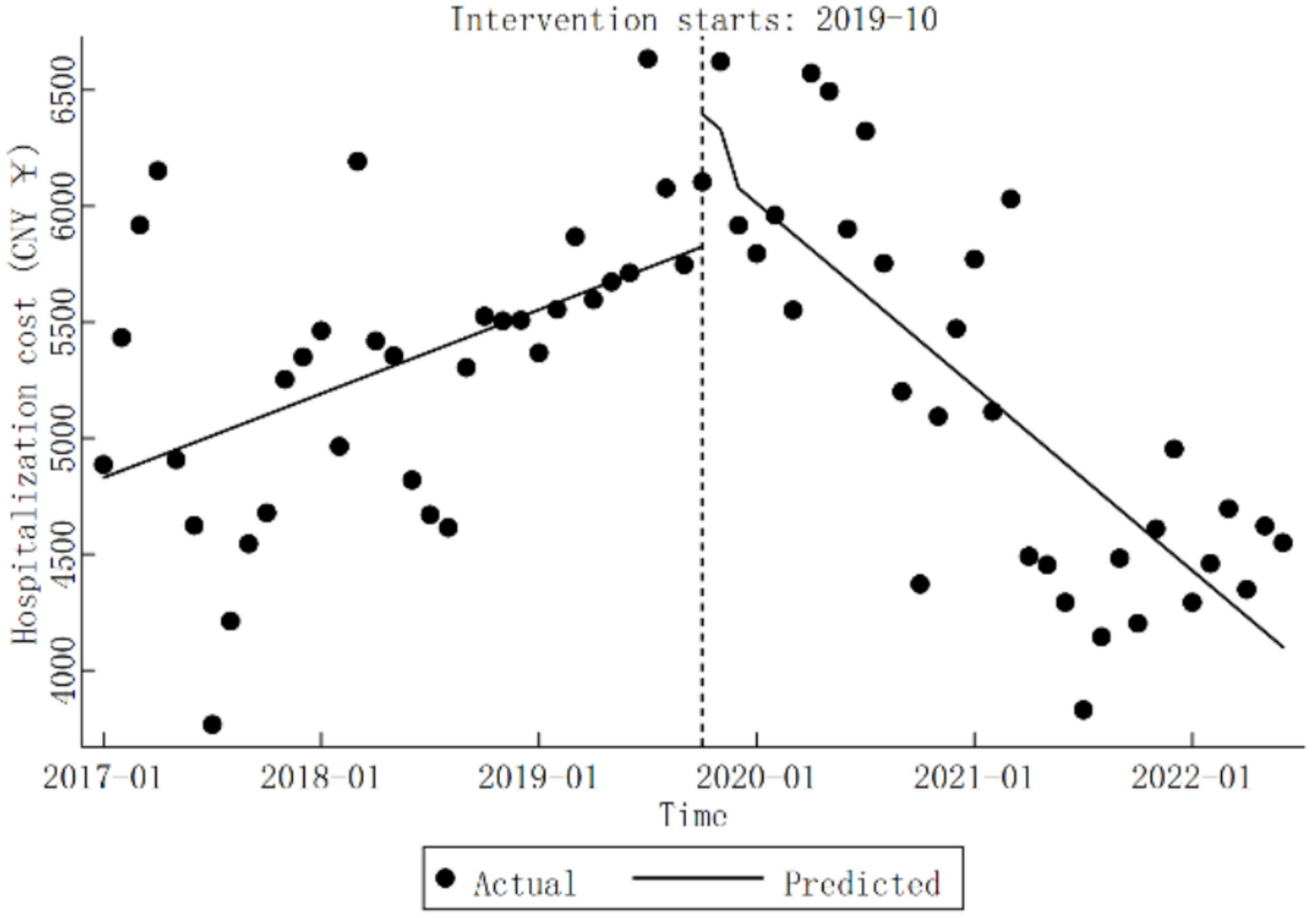
Trends in hospitalization cost for diabetic patients pre- and post-DRG reform.

At Qingyang City Hospital of Chinese Medicine, hospitalization cost exhibited a significant upward trend before the implementation of the DRG reform (*β*_1_ = 30.13, *p* < 0.05). During the reform period, changes in hospitalization cost level were not statistically significant (*β*_2_= 571.48, *p* > 0.05). However, a noteworthy downward trend was observed following the reform, with a monthly average decrease of CNY 67.84 (*β*_1_ + *β*_3_ =−65.91, *p* < 0.05). Detailed results are presented in Table 3, and the specific trend changes are illustrated in Figure 2.

**Table 3:** Results of ITS analysis on healthcare-related costs for diabetic patients.

Items	Value	Std. Err.	*t* value	*P* value	95% conf. interval
Hospitalization cost, CNY					
Baseline level, *β*_0_	4830.49	308.62	15.65	<0.001	4213.36 to 5447.62
Baseline trend, *β*_1_	30.13	13.88	2.17	0.034	2.37 to 57.89
Level change, *β*_2_	571.48	241.62	2.37	0.021	88.34 to 1054.62
Trend change, *β*_3_	−96.04	17.10	−5.61	<0.001	−130.24 to −61.83
Late trend, *β*_1_ + *β*_3_	−65.91	9.99	−6.60	<0.001	−85.88 to −45.93
TCM treatment cost, CNY					
Baseline level, *β*_0_	507.87	70.09	7.25	<0.001	367.73 to 648.02
Baseline trend, *β*_1_	1.56	5.37	0.29	0.772	−9.18 to 12.30
Level change, *β*_2_	−414.09	120.53	−3.44	0.001	−655.11 to −173.07
Trend change, *β*_3_	−2.35	5.38	−0.44	0.664	−13.10 to 8.40
Late trend, *β*_1_ + *β*_3_	−0.78	0.24	−3.23	0.002	−1.27 to −0.30
Chinese medicine cost, CNY					
Baseline level, *β*_0_	594.91	77.29	7.70	<0.001	440.37 to 749.46
Baseline trend, *β*_1_	−4.50	3.26	−1.38	0.173	−11.02 to 2.02
Level change, *β*_2_	204.46	66.12	3.09	0.003	72.23 to 336.68
Trend change, *β*_3_	−1.46	6.13	−0.24	0.813	−13.72 to 10.80
Late trend, *β*_1_ + *β*_3_	−5.95	5.19	−1.15	0.256	−16.33 to 4.42
Western medicine cost, CNY					
Baseline level, *β*_0_	1970.99	356.02	5.54	<0.001	1259.09 to 2682.89
Baseline trend, *β*_1_	−13.91	15.61	−0.89	0.376	−45.12 to 17.30
Level change, *β*_2_	284.68	187.95	1.51	0.135	−91.14 to 660.51
Trend change, *β*_3_	−19.83	16.12	−1.23	0.223	−52.06 to 12.40
Late trend, *β*_1_ + *β*_3_	−33.74	4.01	−8.41	<0.001	−41.76 to −25.72

**Figure 3:**
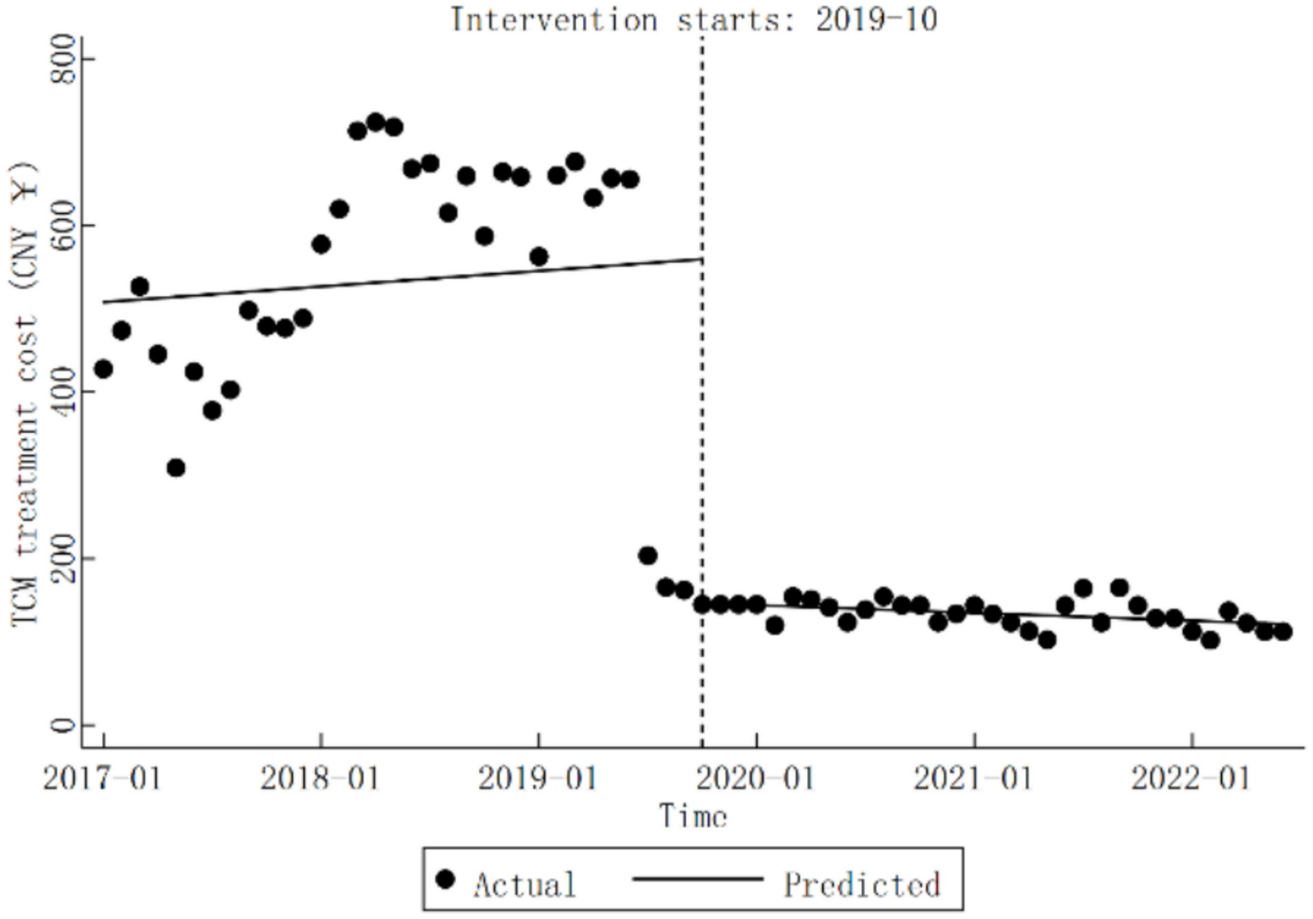
Trends in TCM treatment cost for diabetic patients pre- and post-DRG reform.

For TCM treatment cost, the trend before the DRG reform was not statistically significant (*β*_1_ =1.56, *p* > 0.05). However, during the reform, there was a significant decrease in TCM treatment cost representing CNY 414.09 (*β*_2_ =−414.09, *p* < 0.05). Following the reform, a notable downward trend was observed, with a monthly average decrease of CNY 0.78 (*β*_1_ + *β*_3_ =−0.78, *p* < 0.05). Detailed results are presented in Table 3, and the specific trend changes are illustrated in Figure 3.

**Figure 4:**
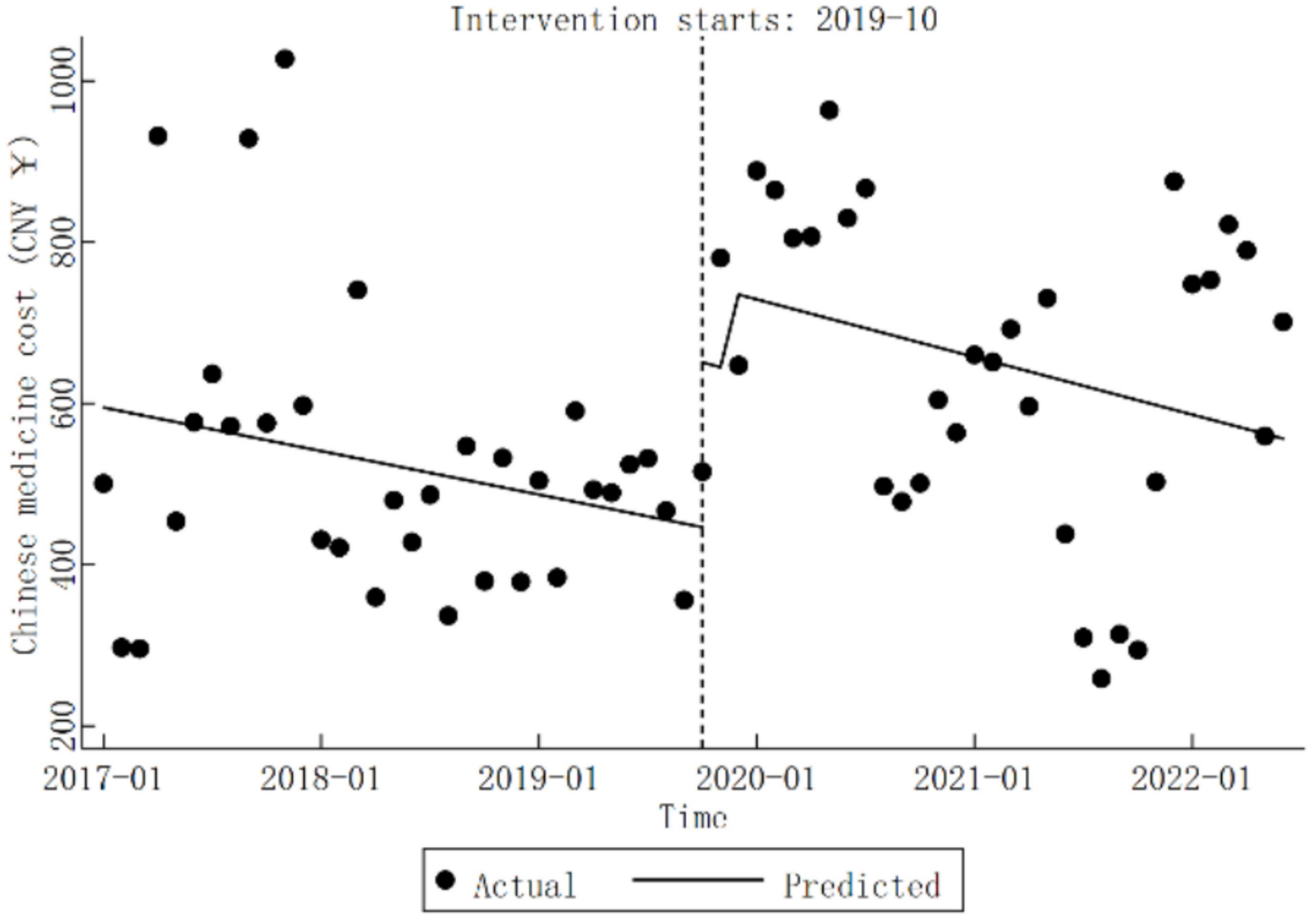
Trends in Chinese medicine cost for diabetic patients pre- and post-DRG reform.

Before the implementation of the DRG reform at Qingyang City Hospital, Chinese medicine cost exhibited an insignificant decline trend (*β*_1_ =−4.50, *p* > 0.05). Conversely, during the reform period, Chinese medicine cost significantly increased by CNY 204.46 (*β*_2_ =204.46, *p* < 0.05). Post-reform, the trend in Chinese medicine cost showed no significant change (*β*_1_ + *β*_3_ =−5.95, *p* > 0.05). Detailed results are summarized in Table 3, with trend changes depicted in Figure 4.

**Figure 5:**
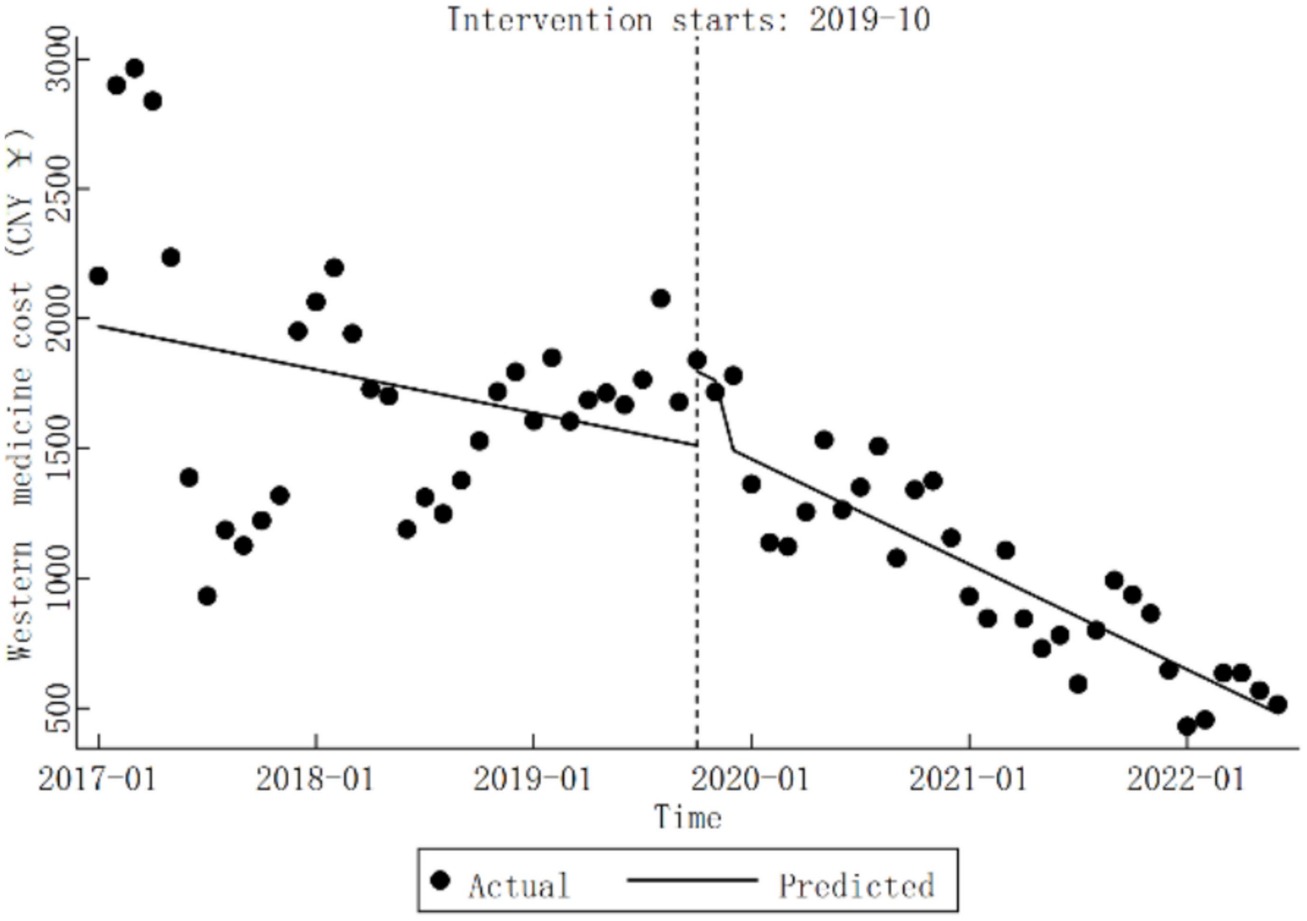
Trends in Western medicine cost for diabetic patients pre- and post-DRG reform.

In comparison, Western medicine cost at Qingyang City Hospital of Chinese Medicine displayed no significant trend pre-DRG reform (*β*_1_ =−13.91, *p* > 0.05). During the reform, Western medicine cost also remained statistically unchanged (*β*_2_ =284.68, *p* > 0.05). However, after the reform, a significant downward trend was observed, with a monthly average decrease of CNY 33.74 (*β*_1_ + *β*_3_ =−33.74, *p* < 0.05). Comprehensive results can be found in Table 3, with trend variations illustrated in Figure 5.

### 3.3 Results of ITS analysis of DRG reform on length of stay at Qingyang City Hospital of Chinese Medicine

We performed a Cumby-Huizinga autocorrelation test on length of stay at Qinyang Hospital. The results revealed that length of stay may exhibit first-order autocorrelation, and we adjusted for autocorrelation by employing the “lag (1)” command to ensure the validity of the ITS analysis, as detailed in Table 4.

**Table 4:** Autocorrelation test results of length of stay for diabetic patients.

*H*_0_: q = 0 (serially uncorrelated)				*H*_0_: q = lag-1			
*H*_1_: s.c. present at range specified				*H*_1_: s.c. present at lag specified			
lags	chi2	df	*P*-value	lags	chi2	df	*P*-value
1–1	3.86	1	0.049	1	3.86	1	0.049
1–2	4.86	2	0.088	2	0.24	1	0.625
1–3	4.86	3	0.182	3	0.15	1	0.702
1–4	5.21	4	0.266	4	0.35	1	0.557
1–5	7.39	5	0.193	5	2.43	1	0.119
1–6	7.78	6	0.255	6	1.28	1	0.259

**Figure 6:**
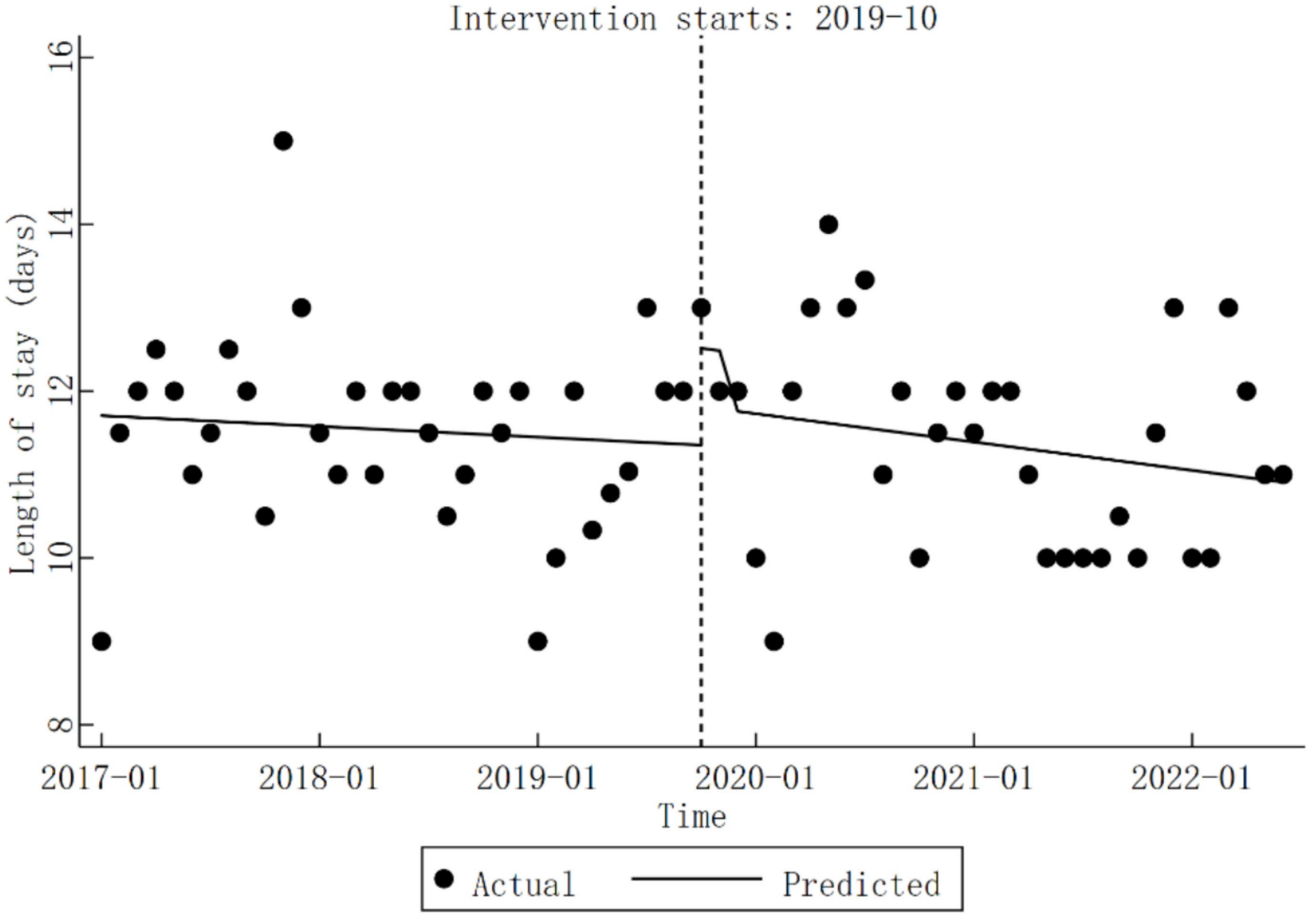
Trends in length of stay for diabetic patients pre- and post-DRG reform.

At Qingyang City Hospital of Chinese Medicine, the trend in inpatient length of stay before the implementation of the DRG reform was not statistically significant (*β*_1_ =−0.01, *p* > 0.05). However, significant changes in length of stay were observed during the reform period (*β*_2_ =1.16, *p* < 0.05). Post-reform, the length of stay did not demonstrate any statistically significant alterations (*β*_1_ + *β*_3_ =−0.03, *p* > 0.05). Detailed results are summarized in Table 5, and specific trend changes are illustrated in Figure 6.

**Table 5:** Results of ITS analysis on length of stay for diabetic patients.

Item	Value	Std. Err.	*t* value	*P* value	95% conf. interval
Baseline level, *β*_0_	11.71	0.46	25.23	<0.001	10.78 to 12.63
Baseline trend, *β*_1_	−0.01	0.02	−0.45	0.657	−0.06 to 0.04
Level change, *β*_2_	1.16	0.48	2.43	0.018	0.20 to 2.12
Trend change, *β*_3_	−0.02	0.04	−0.43	0.667	−0.10 to 0.06
Late trend, *β*_1_ + *β*_3_	−0.03	0.03	−0.86	0.392	−0.09 to 0.04

## 4 Discussion

This study represents the first empirical investigation in Western China to assess the impact of the DRG reform on healthcare-related cost and length of stay for diabetic patients in TCM hospitals. Our findings provide significant insights into controlling costs for TCM-dominant diseases, such as diabetes, through health insurance payment reforms. Within this research, the univariate analysis revealed notable differences in patient demographics and clinical characteristics before and after the reform, particularly in terms of age, visit time, type of diabetes, and complications and comorbidities. Post-reform, we observed that hospitalized patients tended to be older, with a higher proportion experiencing multiple hospitalizations, especially among those with type 2 diabetes. This indicates that the DRG reform has enhanced the appeal of TCM therapies for older patients and those primarily affected by type 2 diabetes. Moreover, type 2 diabetes, as the predominant form of diabetes in China, benefits from relatively standardized and mature clinical pathways, rendering it more responsive to favorable policy adjustments. The cost containment achieved through the DRG reform may have further motivated proactive treatment-seeking behaviors among patients with type 2 diabetes, and in some cases, encouraged repeated hospital admissions as a means to improve health outcomes. However, the observed increase in hospitalization frequency warrants close scrutiny for potential instances of “readmission splitting” —the practice of dividing a single episode of care into multiple admissions—to safeguard the financial integrity of the medical insurance system.

Following the reform, the majority of admitted patients did not present with comorbidities, and there was a marked decrease in surgeries and operations. The DRG reform imposed stricter requirements on medical institutions regarding cost control. In clinical practice, when diagnosing patients with mild chronic conditions, physicians may demonstrate a preference for assigning them to “without complication or comorbidity” and “non-surgical/non-procedural” groups. This strategic classification aimed to achieve more favorable medical insurance reimbursement rates while avoiding the excessive costs associated with more complex DRG classifications. It also served to circumvent the ensuing stringent insurance oversight and potential financial penalties linked to performance evaluations. Addressing this behavioral tendency represents a critical challenge that medical institutions must confront in the next phase of enhancing operational capacity and advancing refined management practices. Notably, our interrupted time-series analysis revealed a significant downward trend in hospitalization cost, TCM treatment cost, and Western medicine cost post-reform, with these metrics remaining lower than their pre-reform levels. In contrast, Chinese medicine cost has demonstrated a consistent trend of gradual adjustment, yet it remains higher than pre-reform level. Simultaneously, length of stay showed a slight, although this change was not statistically significant.

Importantly, the DRG reform has successfully lowered overall hospitalization cost for diabetic patients, consistent with findings from various studies demonstrating its effectiveness in lowering inpatient expenses (34–36). A detailed analysis of hospitalization expense components reveals that TCM hospitals primarily achieve cost reductions by consistently lowering Western medicine cost and TCM treatment cost. However, its effectiveness in managing Chinese medicine cost and length of stay appears limited. The increase in Chinese medicine cost is driven by two primary factors. First, strong governmental support for the development of TCM, including the continuation of the drug markup policy, has created financial headroom for cost growth. Second, TCM’s proven efficacy in managing chronic diseases such as diabetes has earned it substantial patient recognition. This recognition constitutes a form of rigid demand; consequently, patients’ willingness to purchase TCM medicine and related services is unlikely to diminish with the implementation of DRG reform. Furthermore, diabetes, as a major chronic disease in China, follows a relatively standardized treatment pathway, leaving limited room for significant short-term improvements in therapeutic outcomes. Consequently, the DRG reform may also have little effect in reducing the length of stays for these patients.

Interestingly, our study observed no absolute declining trend in length of stay following the reform, which stands in contrast to findings from some studies on DRG insurance reform (37–39). Moreover, while existing research identifies length of stay as a critical factor influencing hospitalization cost (40–42)—implying that a significant cost reduction should coincide with a corresponding decrease in length of stay—our findings diverge from this expectation. We believe that this discrepancy may be attributed to the unique diagnostic and therapeutic approaches inherent to TCM, where effects are typically more gradual compared to those observed in Western medicine. For this reason, relying solely on improving TCM efficacy to reduce length of stay while simultaneously decreasing Western medicine usage has limited effectiveness, although we did observe a slight downward trend in length of stay following the DRG reform. Additionally, TCM is relatively cost-effective, and treatments for chronic diseases usually require longer durations. As patients spend more time hospitalized for TCM treatment, their marginal cost decreases; thus, an increase in length of stay for certain chronic disease patients does not necessarily lead to a sharp rise in hospitalization cost. Conversely, a reduction in hospitalization cost does not directly result in a significant decrease in length of stay. Therefore, the relatively stable trends in length of stay before and after the DRG reform suggest that patients are receiving sufficient TCM services to ensure treatment efficacy. Meanwhile, the notable decline in hospitalization cost post-reform is advantageous for patients, indicating that TCM hospitals have effectively mitigated the economic burden of diabetes on patients while ensuring therapeutic effectiveness through the DRG reform.

In conclusion, the DRG payment reform in TCM hospitals aims to leverage the advantages of TCM in addressing the treatment needs of diabetes, thereby alleviating the economic burden of the disease on patients. However, it is essential to further deepen the reforms in health insurance payments by strengthening cost control mechanisms and implementing various measures to support the inheritance and innovative development of TCM (35, 43, 44). TCM hospitals should consider providing specialized diagnostic and therapeutic services tailored to the needs of chronic diseases such as diabetes, incorporating approaches like acupuncture, herbal prescriptions, and massage therapy to deliver high-quality healthcare services (45–49). This strategy could effectively reduce length of stay and healthcare-related costs while ensuring optimal treatment outcomes.

## 5 Limitations

Our study investigates diabetic inpatients at Qingyang City Hospital of Chinese Medicine, Gansu Province. Although the current sample size is limited and warrants expansion in future research, this hospital is notably the first tertiary TCM institution in Western China to implement DRG payment reform, thereby providing significant representativeness to our findings. Moreover, while the study primarily addresses hospitalization and medication costs, it lacks assessments of healthcare service effectiveness. This limitation is largely due to the underdeveloped medical record systems in TCM hospitals, which should be improved alongside future updates to the case information collection system. Additionally, this study faces challenges in isolating the effects of other health policies implemented during the DRG reform period, as well as the influence of differentiated patient characteristics before and after the reform, on diabetes-related hospitalization costs and length of stay. Future research will seek to quantify these policy interventions or refine the analytical models to enable a more rigorous assessment of the impact of TCM-specific DRG reform.

## 6 Conclusion

The implementation of DRG reform has resulted in a notable decline in hospitalization cost, TCM treatment cost, and Western medicine cost among diabetic patients in TCM hospitals. Conversely, Chinese medicine cost has persisted in following pre-reform trend, but the overall level of cost is higher after the reform than before. Moreover, the average length of stay has exhibited a slight reduction post-reform. To optimize outcomes, TCM hospitals should further advance DRG payment reform, harness the distinctive advantages of TCM, and intensify cost control measures, thereby reducing hospital stays and alleviating the economic burden of illness on patients.

## Data Availability

The original contributions presented in the study are included in the article/Supplementary material, further inquiries can be directed to the corresponding author.
